# miR-1236 down-regulates alpha-fetoprotein, thus causing PTEN accumulation, which inhibits the PI3K/Akt pathway and malignant phenotype in hepatoma cells

**DOI:** 10.18632/oncotarget.3338

**Published:** 2015-01-21

**Authors:** Rui Gao, Chunli Cai, Jiancheng Gan, Xi Yang, Zeyu Shuang, Min Liu, Shengping Li, Hua Tang

**Affiliations:** ^1^ Tianjin Life Science Research Center and Basic Medical School, Tianjin Medical University, Tianjin, China; ^2^ Department of Surgery, Secondary Hospital of Tianjin Medical University, Tianjin, China; ^3^ State Key Laboratory of Oncology in Southern China, Department of Hepatobiliary Oncology, Cancer Center, Sun Yat-sen University, Guangzhou, China

**Keywords:** microRNA, microRNA-1236, AFP, PTEN, hepatocellular cancer

## Abstract

Alpha fetoprotein (AFP) is a clinical biomarker of hepatocellular carcinoma (HCC). Here, we found that miR-1236 is down-regulated, whereas AFP is highly expressed in HCC tissues and cells. We demonstrated that miR-1236 directly targets the 3′UTR of AFP and down-regulates its expression. Also, miR-1236 inhibited and AFP stimulated proliferation, migration, invasion and vasculogenic mimicry (VM) of HCC. In agreement, AFP over-expression counteracted the inhibitory effect of miR-1236. We demonstrated that AFP promoted the ubiquitination of PTEN, thus decreasing PTEN levels, while miR-1236 inhibited the PI3K/Akt pathway.

## INTRODUCTION

Hepatocellular carcinoma (HCC) is one of the most common and aggressive human malignancies with poor prognosis worldwide [1]. HCC occurs more often in men than in women and has become a leading cause of cancer-related death [2]. Most HCC cases are diagnosed in an advanced stage, which is a sub-optimal scenario for surgical resection. Additionally, the treatment options for advanced HCC are limited and ineffective, which contributes to the poor prognosis [3]. There is an urgent need to develop optimize strategies for the early diagnosis and therapy of HCC.

Alpha fetoprotein (AFP) is an oncofetal protein that is normally produced in the fetal liver and yolk sac. AFP is undetectable or present only at trace amounts (<20 ng/ml) in adults but is re-expressed in approximately 70% of HCCs; therefore, AFP is a widely used biomarker for HCC surveillance in clinical settings [4]. High serum AFP levels are positively correlated with HCC size, vascular invasion and poor differentiation [5]. Recent reports showed that serum AFP acts as an immune regulator and may have utility in T cell-mediated immunotherapy [6]. Our previous findings demonstrated that AFP promotes the growth of HCC cells *in vitro* and *in vivo* due to its facilitation on the G1/S transition of the cell cycle [7]. Recent reports have indicated that cytoplasmic AFP colocalizes and interacts with phosphatase and tensin homologue deleted on chromosome 10 (PTEN) and down-regulates PTEN expression while synchronously promoting the PI3K/Akt pathway [8]. The PI3K/Akt signaling pathway is involved in the regulation of cell survival, proliferation, metabolism, migration and vasculogenic mimicry (VM) through modulating numerous substrates via activated AKT [9, 10]. PTEN acts as a phosphatase for PI3P and negatively regulates the PI3K/Akt pathway. The effect of AFP on the PI3K/Akt pathway (mediated by PTEN) contributes to hepatocellular growth and tumorigenesis. Mounting studies on AFP are about its' correlations with the clinical features of HCC. While the biological roles of AFP other than proliferation on the HCC cells is yet to be determined.

The machenism of up-regulation of AFP in HCC is not fully understood. Previous evidence has shown the re-expression of AFP is inhibited at the transcriptional level by p53 [11], β-catenin [12] and ZBTB20 [13]. However, whether AFP is regulated at the post-transcriptional level by microRNA is unknown.

miRNAs are small noncoding RNAs of ~22 nt that regulate gene expression through suppressing protein translation and promoting the degradation of the target mRNAs [14]. miRNAs are estimated to regulate the translation of more than 60% of protein-coding genes [15]. Increasing evidence has demonstrated that the dysregulation of miRNAs is linked to the development of various types of human diseases including cancer. miRNAs may act as oncogenes or tumor suppressors to regulate cell proliferation, apoptosis, migration, invasion and VM [16-18]. Recently, our laboratory and other research groups showed that some miRNAs, such as miR-10a, miR-490-3p and miR-371-5p, miR-142-3p and miR-137 are involved in HCC development by regulating different targets [19-23]. However, it is unknown whether there are miRNAs that directly regulate the expression of AFP. To explore this issue, we predicted miRNAs targeting the 3′ UTR of AFP mRNA and chose miR-1236 for further study.

In the current study, we found that miR-1236 contributes to the high levels of AFP in HCC by directly targeting the 3′ UTR of AFP mRNA and down-regulating its expression. Furthermore, we demonstrated that high levels of AFP promote the proliferation, migration, invasion and VM in HCC cells. Additionally, miR-1236 suppresses the growth of HCC *in vitro* and *in vivo* and also inhibits the migration, invasion and VM by down-regulating the expression of AFP. Moreover, we demonstrated that AFP promotes the ubiquitination of PTEN and accelerates PTEN degradation. Thus, miR-1236 inhibits the PI3K/Akt pathway through the up-regulation of PTEN expression levels by decreasing AFP-mediated repression.

## RESULTS

### miR-1236 directly down-regulates the expression of AFP

To determine whether there are miRNAs that directly regulate the expression of AFP, we predicted the potential miRNAs that target AFP using the prediction algorithms of TargetScan, PicTar and miRanda. In all the miRNAs that were predicted, miR-1236 was chosen for further study according to its high scores.

**Figure 1:**
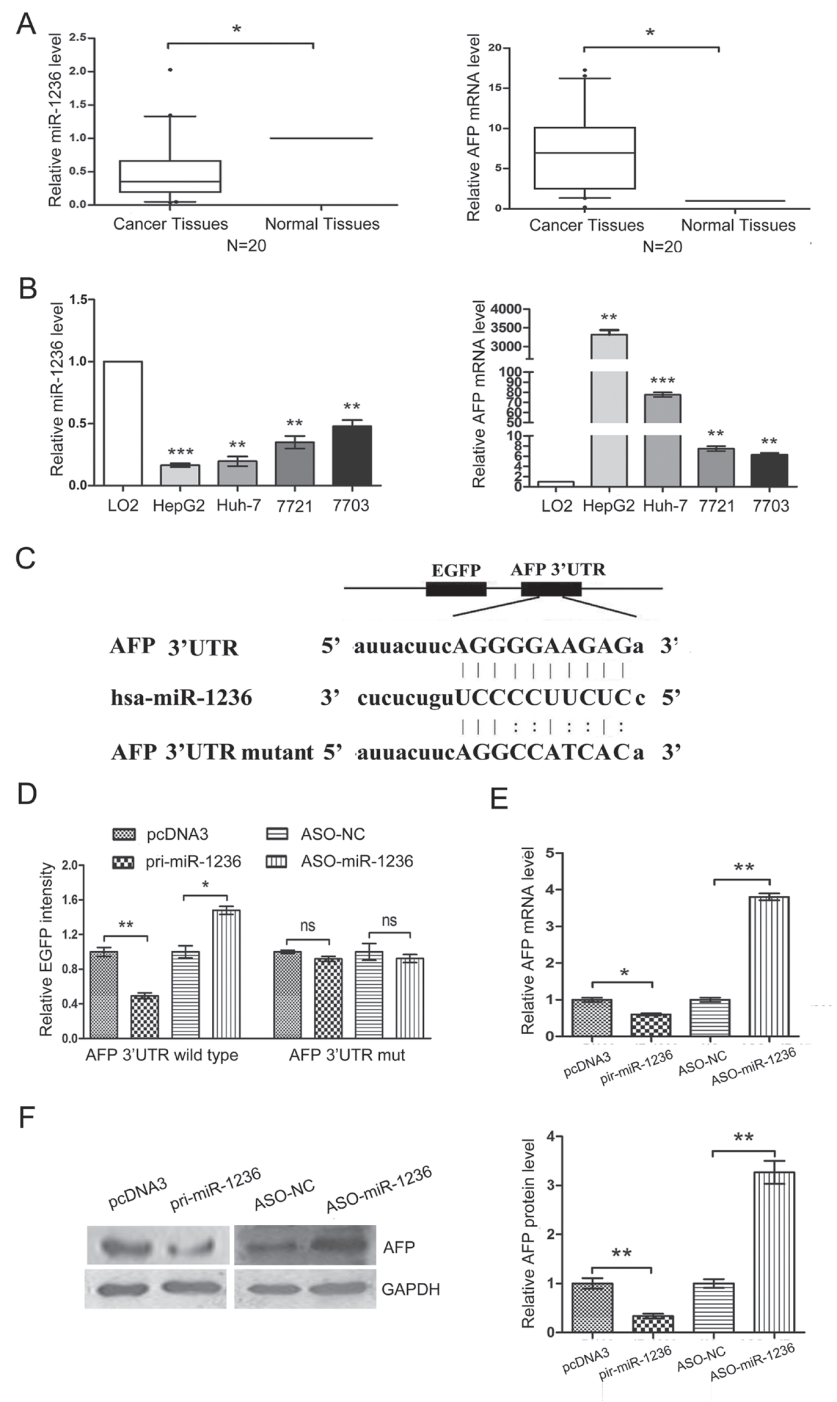
miR-1236 directly down-regulates the expression of AFP (A) The expression levels of miR-1236 (Left) and AFP (Right) mRNA in 20 pairs of HCC tissues and adjacent non-tumor tissues were detected by qRT-PCR. U6 and β-actin were used as internal controls to normalize the levels of miR-1236 and AFP, respectively. (B) qRT-PCR was used to detect the expression levels of miR-1236 (Left) and AFP (Right) mRNA in LO2, HepG2, Huh-7, SMMC-7721 and QGY-7703 cells. U6 and β-actin were used as internal controls to normalize the levels of miR-1236 and AFP, respectively. (C) The predicted binding sites for miR-1236 in the 3′UTR of AFP and the mutations in the binding sites are shown. (D) The EGFP reporter assay was performed in QGY-7703 cells cotransfected pcDNA3/EGFP-AFP 3′UTR wild type or pcDNA3/EGFP-AFP 3′UTRmut with pri-miR-1236 or ASO-miR-1236. (E) qRT-PCR was performed to detect the AFP mRNA level in QGY-7703 cells transfected with pcDNA3/pri-miR-1236, ASO-miR-1236 or the corresponding controls. (F) Western blot assays were used to detect the AFP protein level in QGY-7703 cells transfected with pcDNA3/pri-miR-1236, ASO-miR-1236 or the corresponding controls (Left), and the quantification of the bars are shown on the right. *p<0.05, **p<0.01, *** p<0.001, ns, no significance. All error bars indicate the means±SDs. All experiments were repeated at least three times.

To confirm whether miR-1236 contributes to high AFP levels in HCC patients, we first examined the expression of miR-1236 and AFP in 20 pairs of HCC tissues and adjacent non-tumor tissues using qRT-PCR. miR-1236 was down-regulated in HCC tissues relative to adjacent non-tumor tissues (Figure 1A Left), while AFP was up-regulated in HCC tissues (Figure 1A Right). Next, we analyzed the expression of miR-1236 and AFP in the human immortalized normal liver cell line LO2 and four HCC cell lines: HepG2, QGY-7703, SMMC-7721 and Huh-7. Consistent with the results of tissues analysis, miR-1236 expression was lower in the four HCC cell lines than in LO2 cells (Figure 1B Left), but the expression of AFP was higher in the four HCC cell lines than in LO2 cells (Figure 1B Right). The expression levels of miR-1236 and AFP appeared to be inverse correlation in tissues and cells, which prompted us to determine whether miR-1236 directly targets the 3′ UTR of AFP.

To confirm AFP is a target of miR-1236, a human AFP 3′ UTR fragment containing the binding sites of miR-1236 or the mutant sites (Figure 1C) was cloned to the EGFP downstream of pcDNA3/EGFP. Next, an EGFP reporter assay was performed to detect whether AFP is a direct target of miR-1236. First, we cotransfected pcDNA3/EGFP-AFP 3′ UTR wild type or pcDNA3/EGFP-AFP 3′ UTRmut with pri-miR-1236 or ASO-miR-1236 and the control vectors into QGY-7703 cells and then detected the EGFP intensity at 48 h. The over-expression of miR-1236 decreased the fluorescent intensity of the wild type AFP 3′ UTR. In contrast, ASO-miR-1236 increased the fluorescent intensity of that (Figure 1D). However, neither the over-expression nor inhibition of miR-1236 affected the fluorescent intensity of the mutant AFP 3′ UTR (Figure 1D).

Next we explored whether miR-1236 influences the expression of endogenous AFP using qRT-PCR and western blotting. The results showed that the over-expression of miR-1236 decreased the AFP mRNA and protein levels, while the blockage of miR-1236 resulted in an increase in the AFP mRNA and protein levels (Figure 1E and F). These results demonstrate that miR-1236 directly targets the 3′ UTR of AFP mRNA and down-regulates its expression.

### miR-1236 inhibits the proliferation of HCC cells *in vitro* and *in vivo* and delays the cell cycle progression

**Figure 2:**
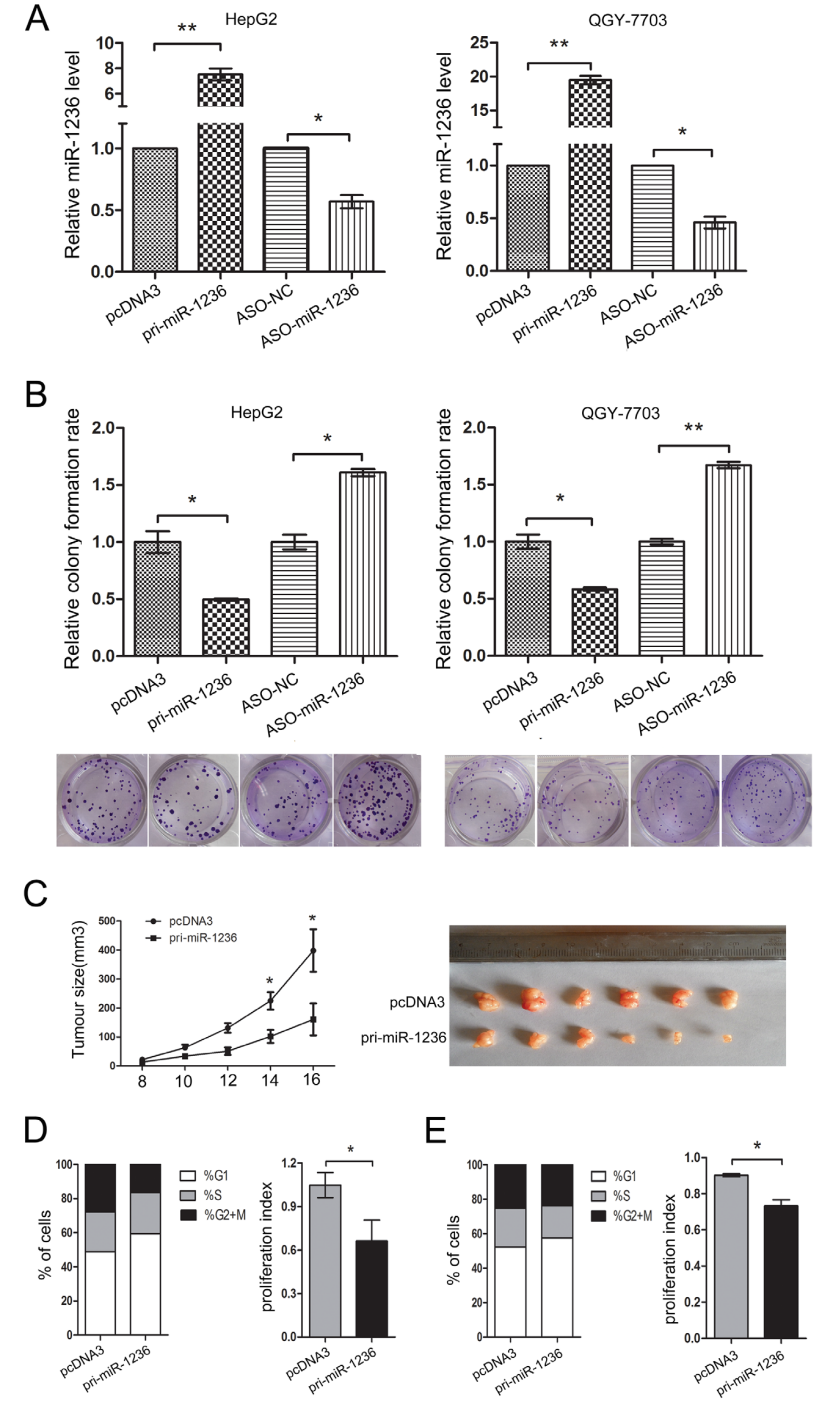
miR-1236 inhibits the proliferation of HCC cells *in vitro* and *in vivo* and delays the cell cycle progression (A) The efficiency of pcDNA3/pri-miR-1236 and ASO-miR-1236 plasmids in HepG2 and QGY-7703. (B) Colony formation assays were performed to test the influence of miR-1236 on the proliferation of HepG2 and QGY-7703 cells. The colony formation rate = (the number of colonies/the number of cells plated) × 100%. (C) The nude mouse tumor xenograft model was utilized to study the effect of miR-1236 on tumor growth *in vivo*. The tumor size was measured 8 days after injection every other day; the tumor growth curve is shown on the left. The tumor volume = length × width^2^×1/2, *p<0.05, n=6 (Student's t test). The mice were euthanized, and the tumors were isolated 16 days after implantation (Right). (D, E) The cell cycle progression of HepG2 (D) and QGY-7703 (E) cells was analyzed by flow cytometry. The chart shows the population of cells in G1-, S- and G2/M–phase (Left) as well as the proliferation index (Right) of the groups transfected with pcDNA3/pri-miR-1236 and pcDNA3. proliferation index = (S + G2/M)/G1 (S, G2/M and G1 are the percentages of cells in S-phase, G2/M-phase and G1-phase, respectively) *p<0.05, **p<0.01. All error bars indicate the means±SDs. All experiments were repeated at least three times.

Before identifying the effect of miR-1236 on the proliferation of HCC cells, the efficiency of pcDNA3/pri-miR-1236 and ASO-miR-1236 in HepG2 and QGY-7703 cells was validated using qRT-PCR (Figure 2A). Then, a colony formation assay was performed. The results indicate that the over-expression of miR-1236 inhibited the colony formation rate both in HepG2 and QGY-7703 cells. Conversely, ASO-miR-1236 increased the colony formation rate in HepG2 and QGY-7703 cells (Figure 2B). To further confirm the inhibitory effect of miR-1236 on tumor growth, we performed animal experiments using a nude mouse tumor xenograft model. As observed in Figure 2C, the average tumor volume was lower in the miR-1236 group than in the control group.

To determine whether miR-1236 regulates the cell cycle, flow cytometry was performed. The results indicate that miR-1236 inhibites the G1/S transition and the proliferation index both in HepG2 (Figure 2D) and QGY-7703 cells (Figure 2E). These results suggest that miR-1236 suppresses the proliferation of HCC cells *in vitro* and *in vivo* by blocking the G1/S transition.

### miR-1236 inhibits the migration, invasion and VM of HCC cells and regulates EMT- and VM-associated molecules

**Figure 3:**
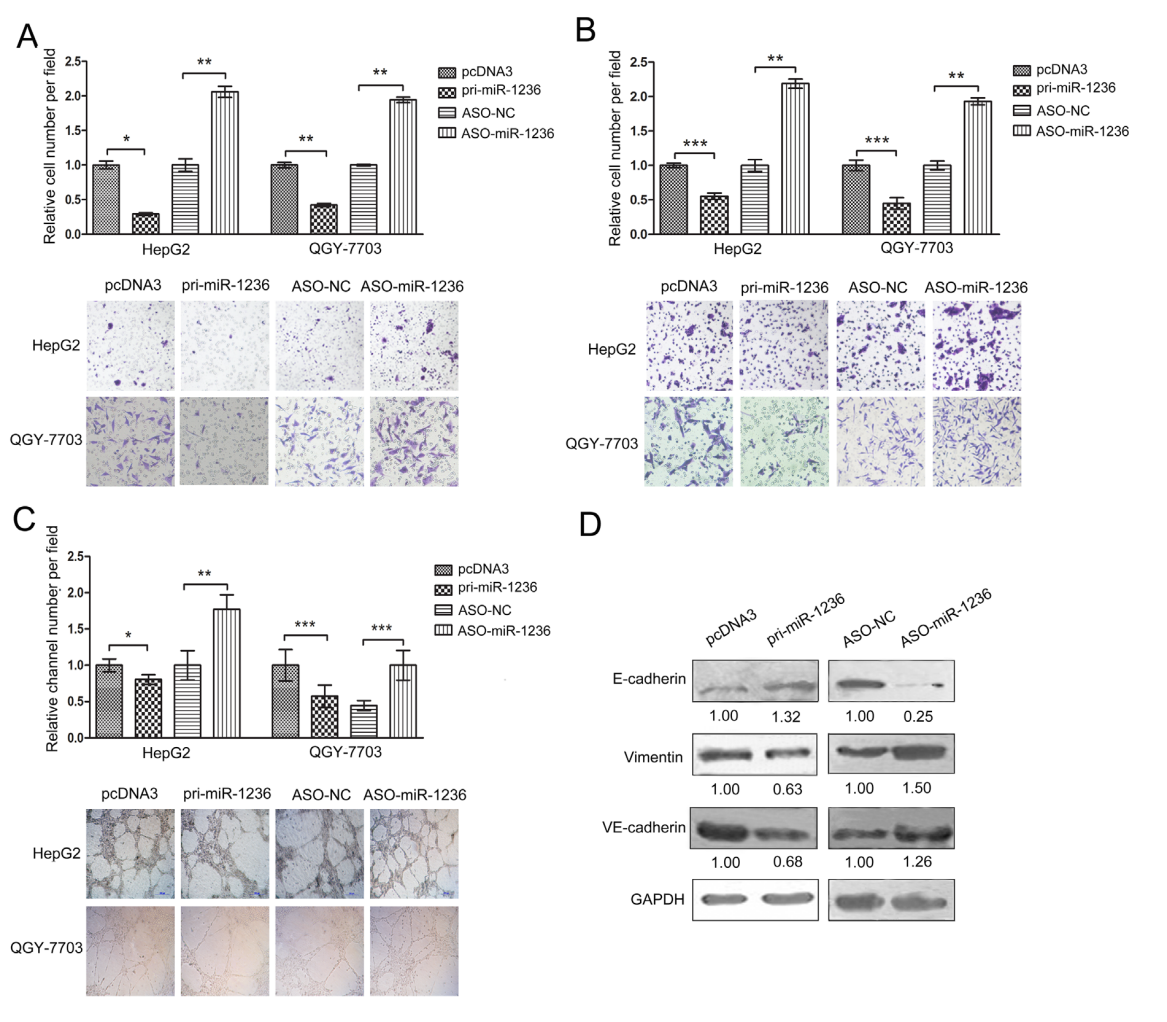
miR-1236 inhibits the migration, invasion and VM of HCC cells and regulates the EMT- and VM-associated molecules (A) Transwell migration assays were performed to detect the migration ability of HepG2 and QGY-7703 cells transfected with pcDNA3/pri-miR-1236, ASO-miR-1236 or the corresponding controls. (B) Transwell (coated with Matrigel) invasion assays were performed to determine the invasion ability of HepG2 and QGY-7703 cells transfected with pcDNA3/pri-miR-1236, ASO-miR-1236 or the corresponding controls. (C) Three-dimensional Matrigel culture assays were performed to detect VM in HepG2 and QGY-7703 cells transfected with pcDNA3/pri-miR-1236, ASO-miR-1236 or the corresponding controls. (D) The influence of miR-1236 on the protein levels of EMT-associated molecules (E-cadherin and vimentin) and of a key VM molecule (VE-cadherin) was determined by western blotting. *p<0.05, **p<0.01, ***p<0.001. All error bars indicate the means±SDs. All experiments were repeated at least three times.

To determine whether miR-1236 influences the migration and invasion of HCC cells, migration and invasion assays were conducted in HepG2 and QGY-7703 cells using transwell chamber inserts. As expected, the over-expression of miR-1236 significantly suppressed the migration and invasion of the HepG2 and QGY-7703 cells, whereas ASO-miR-1236 increased the migration and invasion of both the HepG2 and QGY-7703 cells (Figure 3A and B).

Solid tumor growth is dependent upon an adequate blood supply. VM, a novel mechanism to supply a solid tumor with blood through vessel-like structures formed by tumor cells, has attracted increasing attentions [24]. VM has been observed in several tumor types including HCC [25]. A VM assay was performed to determine whether miR-1236 influences VM in HCC cells. We found that the over-expression of miR-1236 inhibites VM. In contrast, ASO-miR-1236 promotes VM in HepG2 and QGY-7703 cells (Figure 3C).

Next, we tested whether miR-1236 affected the expression of key molecular markers in EMT (E-cadherin and vimentin) and VM (VE-cadherin). As shown in Figure 3D, the over-expression of miR-1236 increased E-cadherin but decreased VE-cadherin and vimentin protein levels in QGY-7703 cells. In contrast, ASO-miR-1236 decreased E-cadherin but increased VE-cadherin and vimentin protein levels in QGY-7703 cells (Figure 3D). These results further indicate that miR-1236 is able to suppress the migration, invasion and VM of HCC cells.

### AFP contributes to the malignant phenotypes of HCC

**Figure 4:**
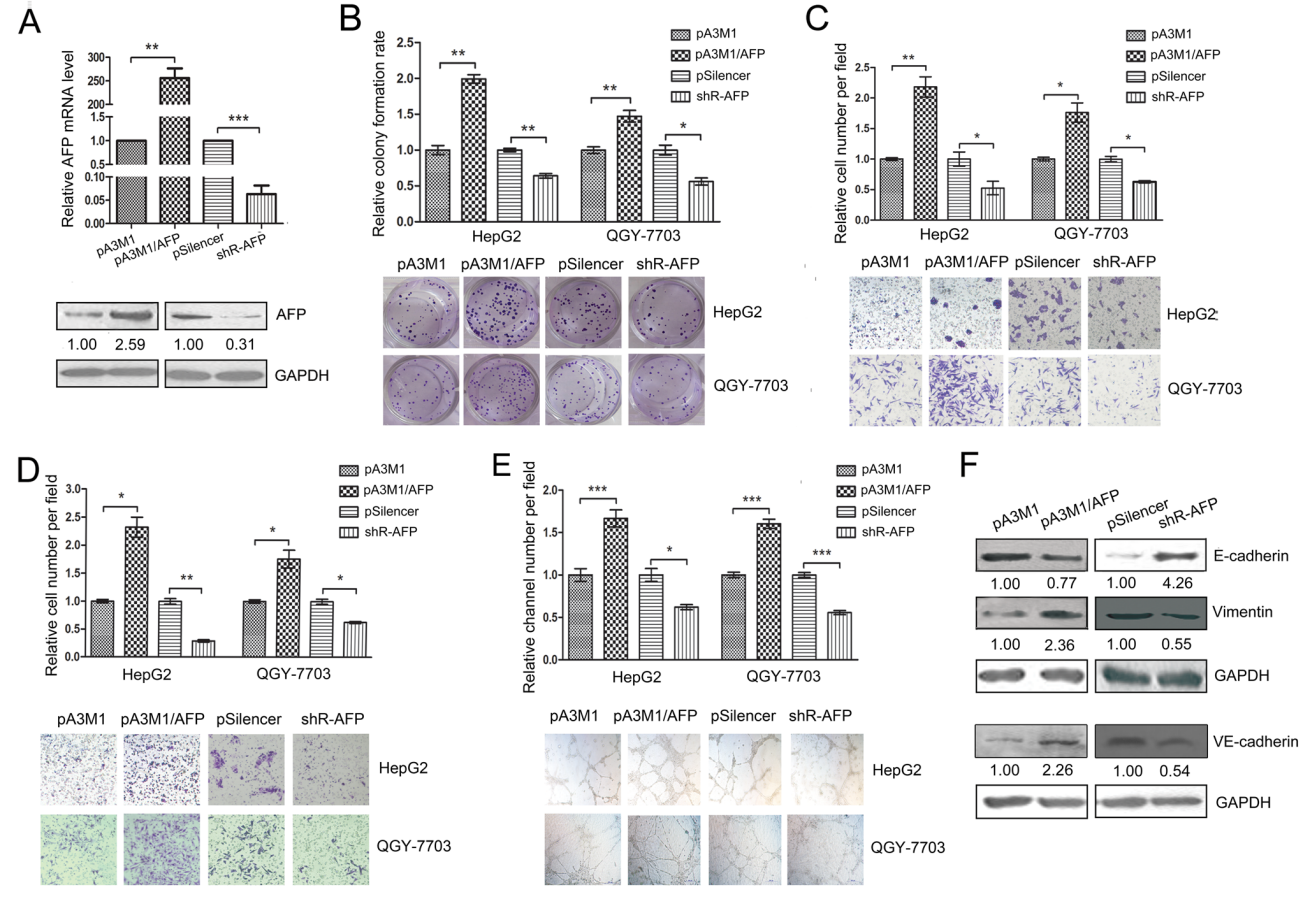
AFP contributes to the malignant phenotypes of HCC (A) The qRT-PCR and western blot were used to test the efficiency of pA3M1/AFP and pSilenser2.1-neo/shR-AFP. (B) Colony formation assays were performed to test the influence of AFP on the proliferation of HepG2 and QGY-7703 cells. (C, D) Transwell migration and invasion assays were performed to detect the effect of AFP on the migration (C) and invasion (D) of HepG2 and QGY-7703 cells. (E) The effect of AFP on the VM in the HepG2 and QGY-7703 cells by means of three-dimensional Matrigel culture. (F) The influence of AFP on the protein levels of EMT-associated molecules (E-cadherin and vimentin) and of a key VM molecule (VE-cadherin) was determined by western blotting. To detect the level of VE-cadherin, the QGY-7703 cells were transfected for 60 h before the protein was harvested. *p<0.05, **p<0.01, ***p<0.001. All error bars indicate the means±SDs. All experiments were repeated at least three times.

To further study the biological roles of AFP in HCC, we constructed an AFP expression plasmid pA3M1/AFP and a knockdown plasmid pSilencer2.1-neo/shR-AFP. The efficiency of the plasmids was validated using qRT-PCR and western blotting (Figure 4A). The colony formation assays of HepG2 and QGY-7703 cells showed that increasing the level of AFP accelerated the proliferation of HCC cells, but the knockdown of AFP reduced the proliferation of HCC cells (Figure 4B). The migration/invasion assays showed that the over-expression of AFP promoted both migration and invasion in these two cell lines. In contrast, decreasing the level of AFP inhibited the migration and invasion (Figure 4C and D). Moreover, we investigated the influence of AFP on VM and found that high levels of AFP promoted VM in HCC cells (Figure 4E).

Furthermore, we used western blotting to investigate the influence of AFP on the specific molecules associated with EMT and VM. The over-expression of AFP down-regulated E-cadherin but increased VE-cadherin and vimentin levels, while the inhibition of AFP expression increased E-cadherin and decreased VE-cadherin and vimentin levels (Figure 4F), which is consistent with the role in the promotion of migration/invasion and VM. In conclusion, AFP may function as an oncogene to promote the proliferation, migration/invasion and VM in HCC.

### The ectopic expression of AFP counteracts the inhibition of the aggressive malignance induced by miR-1236

**Figure 5:**
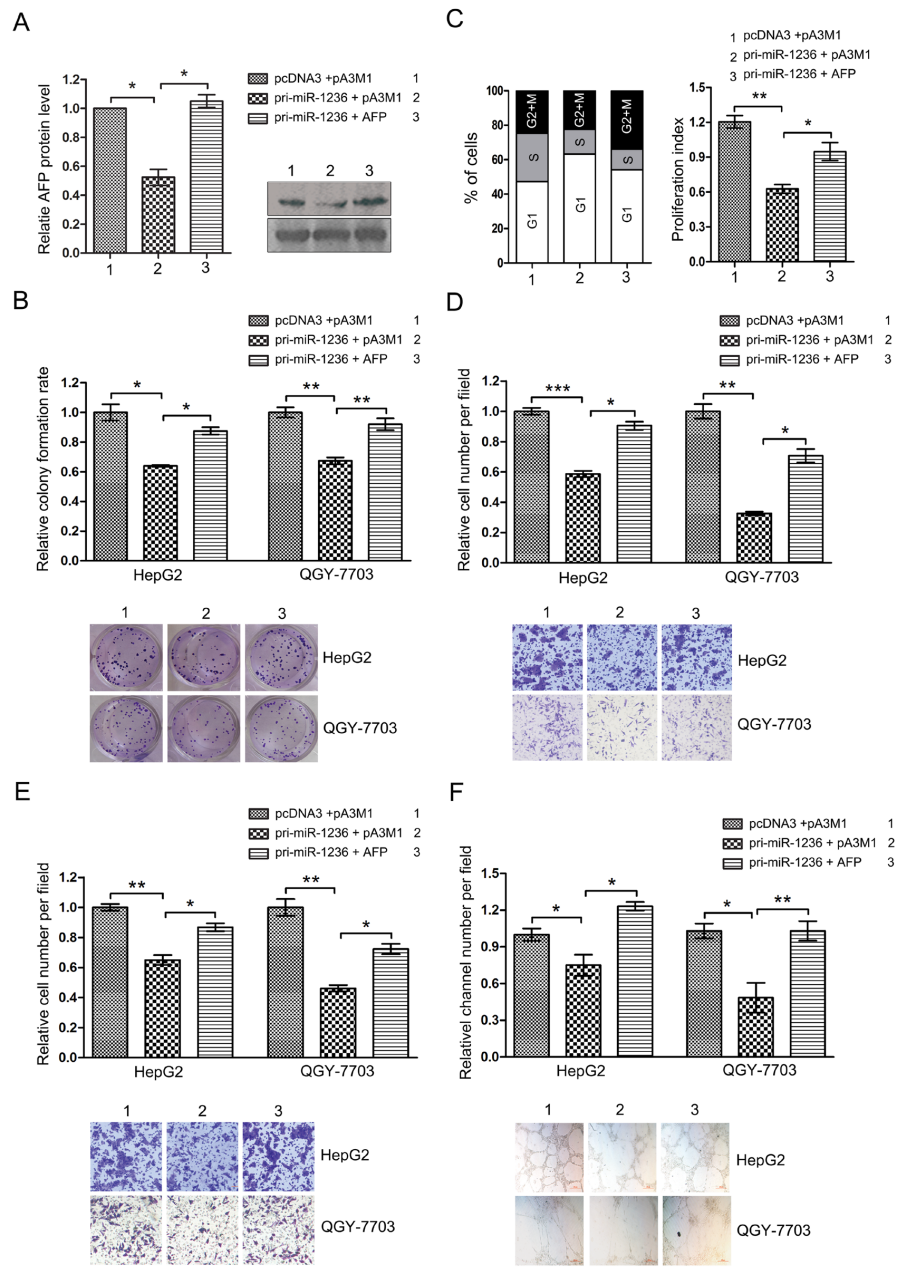
The ectopic expression of AFP counteracts the inhibition of the aggressive malignance induced by miR-1236 (A) HepG2 and QGY-7703 cells were cotransfected with pcDNA3/pri-miR-1236 and pA3M1/AFP without its 3′UTR or the control vector and then western bolt assay was used to test the restoration of AFP protein by pA3M1/AFP in the presence of miR-1236 (Right). The quantification of the bars are shown on the left. (B) The transfected cells were submitted to colony formation assays to test the proliferation of HCC cells. (C) Cell cycle progression of the transfected cells was analyzed by flow cytometry. The left side of the chart shows populations of cells in the different phases of the cell cycle, and the right side shows the proliferation index. (D-F) Transwell migration/invasion assays and three-dimensional Matrigel culture to test the cells' abilities to migrate (D), invade (E) and undergo VM (F). *p<0.05, **p<0.01, ***p<0.001. All error bars indicate the means±SDs. All experiments were repeated at least three times.

The above observations indicate that miR-1236 and AFP have opposite effects on the aggressive phenotypes of HCC cells and that miR-1236 down-regulates AFP expression. Based on these results, we questioned whether the effect of miR-1236 on HCC cells is mediated by the miR-1236 down-regulatory effect on AFP expression. We performed a rescue assay to address this issue. First, we cotransfected miR-1236 with the AFP expression plasmid without the 3′ UTR and confirmed the over-expression of AFP could rescue the decrease in AFP protein levels caused by miR-1236 (Figure 5A). We then performed different functional rescue experiments. As expected, the restoration of AFP expression mostly blocked the inhibitory influence of miR-1236 on the clony formation rate (Figure 5B). In addition, the restoration of AFP can also counteract the suppression of the G1/S transition via miR-1236 (Figure 5C). Meanwhile, the ectopic expression of AFP counteracts the inhibition of migration/invasion and VM induced by miR-1236 in HCC (Figure 5D, E, and F).

### miR-1236 regulates the PTEN/PI3K/Akt pathway mediated by AFP

**Figure 6:**
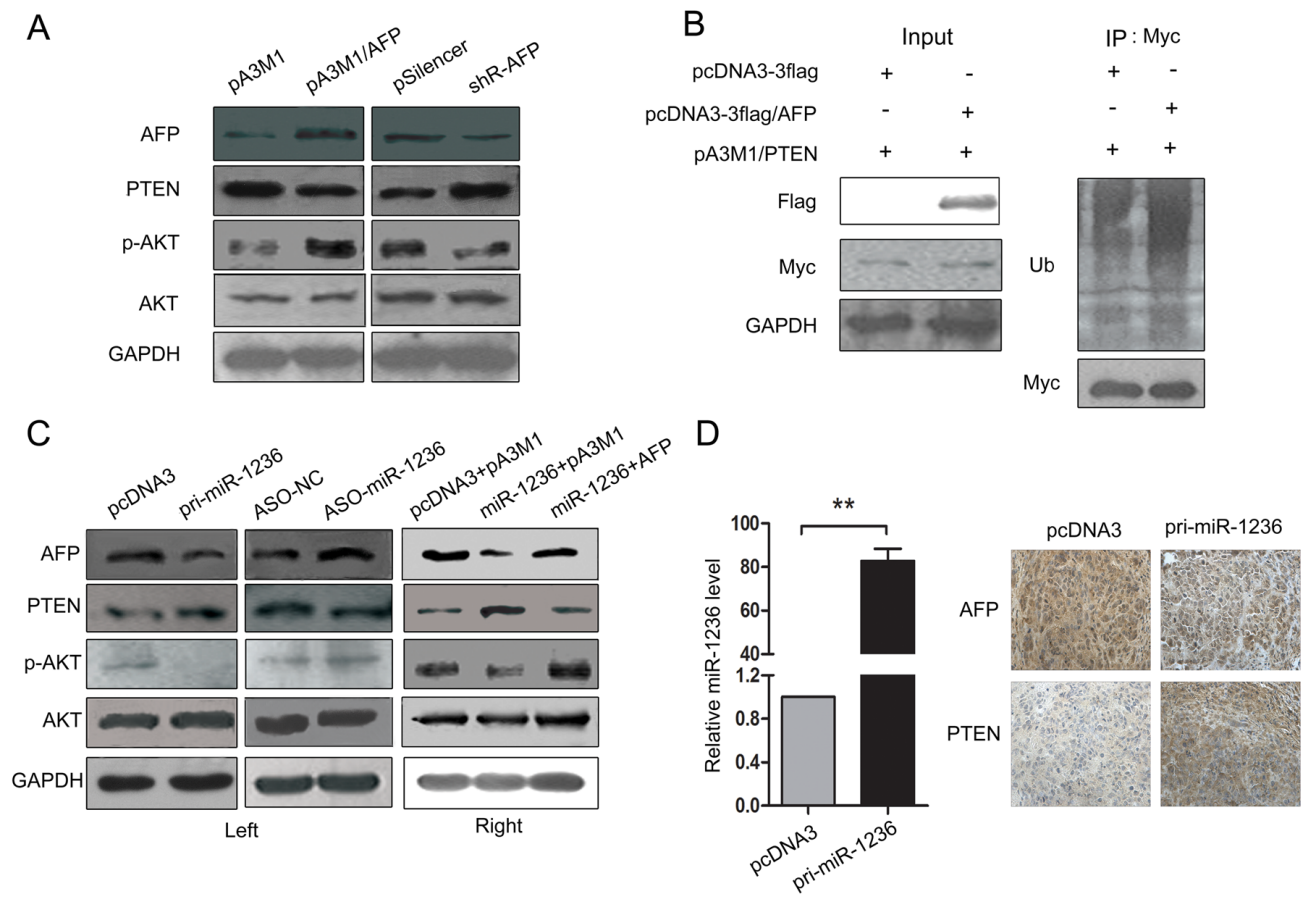
miR-1236 regulates the PTEN/PI3K/Akt pathway mediated by AFP (A) Western blot assays were performed to determine the effect of AFP on the expression of PTEN, p-AKT and AKT in QGY-7703 cells. (B) 293T cells were cotransfected with pA3M1/PTEN (Myc tag) and pcDNA3-flag or pcDNA3-flag/AFP (Flag tag) and then being treated as described in the materials and methods, and the influence of ectopic AFP expression on the ubiquitination levels of PTEN was determined by western blotting. (C) Western blotting was performed to determine the effect of miR-1236 on the expression of AFP, PTEN, p-AKT and AKT in QGY-7703 cells (Left). Western blot assays were performed to detect the restoration of AFP upon presentation of miR-1236 as well as to determine the influence on the expression of PTEN, p-AKT and AKT in QGY-7703 cells (Right). (D) The expression level of miR-1236 in tumor tissues isolated from the nude mice described in Figure 2C (Left). Representative IHC images showing the magnitude of AFP and PTEN expression in tissues isolated from nude mice (Right). **p<0.01, n=3. All error bars indicate the means ± SDs.

AFP contributes to the malignance of HCC, but the mechanism is poorly understood. AFP may be involved in the PI3K/Akt signaling pathway [26], and PTEN, a tumor suppressor, is a negative regulator in this pathway [27]. Here, we found the over-expression of AFP decreased PTEN expression and thus promoted the PI3K/Akt pathway, but the knockdown of AFP increased PTEN levels and resulted in the inhibition of the PI3K/Akt pathway (Figure 6A). Furthermore, a ubiquitination analysis *in vivo* indicated that AFP over-expression promoted the ubiquitination of PTEN (Figure 6B), which influences PTEN stability. Our results revealed that AFP is a direct target of miR-1236 and decreases PTEN levels by promoting PTEN ubiquitination. Therefore, we speculated that miR-1236 may inhibit the PI3K/Akt pathway by up-regulating the expression of PTEN (via decreasing AFP levels). Next we verified the influence of miR-1236 on the protein levels of PTEN and p-AKT. The results showed high levels of miR-1236 increases the expression of PTEN while decreases the phosphorylation of AKT (Figure 6C Left), which demonstrates that miR-1236 inhibits the PI3K/Akt pathway. To confirm that the effect of miR-1236 on the PI3K/Akt pathway is mediated by AFP, we similarly cotransfected pcDNA3/pri-miR-1236 and the AFP expression plasmid without the 3′ UTR and found that AFP can rescue the suppression of PI3K/Akt pathway caused by miR-1236 (Figure 6C Right). To further assess the regulatory effect of miR-1236 on the PTEN/PI3K/Akt pathway, we chosen three pairs of tumors isolated from nude mice as described in Figure 2C, and performed immunohistochemistry (IHC) to detect the magnitude of AFP and PTEN expression. The expression of miR-1236 in these tissues was also investigated using qRT-PCR (Figure 6D Left). The results from the tissues IHC further demonstrated that miR-1236 inhibits AFP expression and promotes PTEN expression (Figure 6D Right). All the results indicate that miR-1236 down-regulates the expression of AFP, thus inhibiting the ubiquitination of PTEN to up-regulate PTEN expression and leading to the inhibition of the PI3K/Akt pathway.

## DISCUSSION

The complex processes of initial and development in HCC are regulated by various factors [28, 29]. It was reported that AFP, KIF18A and KIAA1114 are biological markers correlated with HCC [30, 31], and AFP is widely used in clinical. The re-expression of AFP in most HCC patients has been studied for decades. AFP transcriptional activity is controlled by five distinct regulatory regions: the promoter [32], the repressor region and three enhancers upstream of the repressor [33]. Transcription factor p53 and ZBTB20 repress AFP expression by directly binding to the core promoter of AFP [11, 13]. Other transcription factors, such as hepatocyte nuclear factor 1 (HNF-1), nuclear factor 1 (NF-1), and CAAT/enhancer binding protein (C/EBP), can recognize specific regions and influence promoter activity. Though the regulation of AFP expression at the transcriptional level has been elucidated, the posttranscriptional regulation remains to be thoroughly described.

miRNAs regulate the expression of target genes at the posttranscriptional level via incomplete complementation with the 3′ UTR of target genes; this results in translation inhibition and decreased mRNAs stability. miRNAs participate in diverse biological processes, such as proliferation, migration, invasion and VM formation; thus, miRNAs are involved in tumorigenesis, including that in HCC. To our knowledge, there are no reports on the regulation of AFP expression at the posttranscriptional levels. In this study, we used the prediction algorithms of TargetScan, PicTar and miRanda to predict that miR-1236 can target the 3′ UTR of AFP. We first used the EGFP reporter system to confirm that miR-1236 directly targets the 3′ UTR of AFP and down-regulates the expression of EGFP. Next, we confirmed that miR-1236 inhibits endogenous AFP expression at both the mRNA and protein levels. Moreover, the expression of miR-1236 was lower in HCC tissues relative to adjacent non-tumor tissues, which is in contrast to the expression of AFP. This finding further supports the assertion that miR-1236 down-regulates the expression of AFP by directly binding to the 3′ UTR of AFP.

High levels of AFP have been used as a diagnostic marker for HCC, but the biological roles of AFP in HCC have only been partially described. AFP may be involved in the initiation and development of HCC. Previous work in our laboratory has shown that AFP promotes the proliferation of HCC cells by facilitating the G1/S transition and regulates the expression of cell cycle controlling proteins, such as SKP2, Cyclin D1, Csk and EBAG9 [7]. In the present study, we showed that (along with its effect on proliferation) AFP promotes other aggressive phenotypes, included migration, invasion and VM in HCC. These results prove that AFP is more than a diagnostic marker, it may also play an oncogenic role and contributes to the development of HCC.

AFP is regulated by miR-1236 and promotes the development of HCC. Up to now, there are no reports on the roles of miR-1236 in HCC. We proposed that miR-1236 may inhibit the aggressive phenotypes by decreasing AFP expression. Consistent with our expectations, miR-1236 inhibited the proliferation, migration, invasion and VM in HCC cells. Importantly, the ectopic expression of AFP without the 3′ UTR mostly rescued the inhibitory effect of miR-1236 on the aggressive phenotypes. These results prove that miR-1236 inhibits the development of HCC and may partially (if not completely) through down-regulating AFP expression.

**Figure 7:**
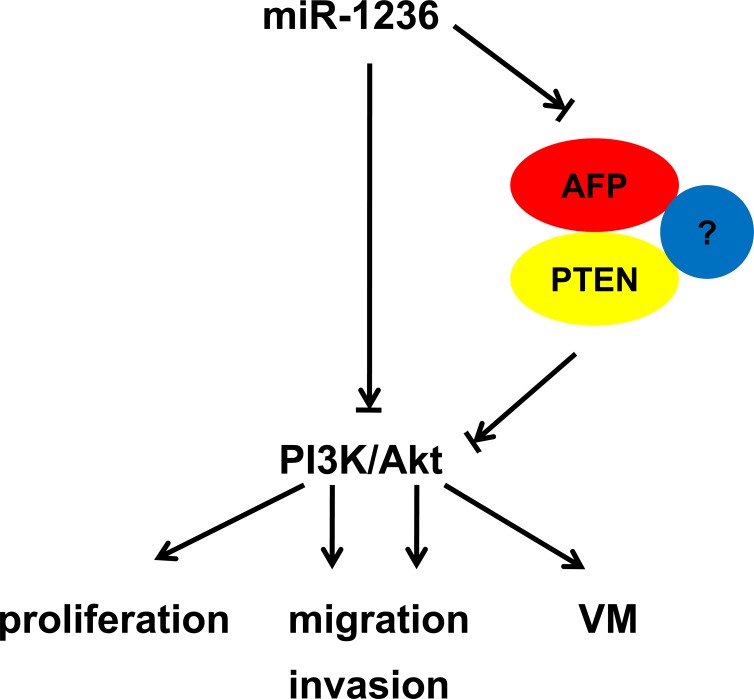
The schematic plot for the regulatory relationship among miR-1236, AFP and PTEN in HCC miR-1236 down-regulates alpha-fetoprotein, thus causing PTEN accumulation, which inhibits the PI3K/Akt pathway and malignant phenotypes including proliferation, migration/invasion and vasculogenic mimicry.

The oncogenic role of AFP in HCC has been confirmed, while the exact mechanism by which AFP influences the development of HCC is known little. A previous study showed that AFP activates the PI3K/Akt pathway through PTEN [8]. The PI3K/Akt signaling pathway is activated in numerous cancers and plays important roles in tumor progression by phosphorylating various substrates such as mTOR, BCL-2, FoxO-family transcription factors, among others [34, 35]. PTEN, acts as a phosphoinositide phosphatase to counteract the effect of PI3K and thus inhibit the PI3K/Akt pathway. The oncogenic role of the PI3K/Akt pathway is well-known, and PI3K inhibitors have been administered as part of human cancer therapy [36, 37]. Thus, the promotion of AFP on the PI3K/Akt pathway contributes to more aggressive HCC phenotypes. However, the basic mechanism of down-regulation of PTEN via AFP is not clear. In the present study, we found that AFP promotes the ubiquitination of PTEN and may accelerate the degradation of PTEN via proteasome. However, it remains unknown which E3 ligases or deubiquitination enzymes of PTEN are influenced by AFP. A previous study showed that AFP interacted with one of E3 ligase (XIAP) of PTEN [38, 39]. We speculate AFP may recruit the E3 ligase (XIAP) to PTEN and lead to the degration of PTEN (Figure 7). Some other E3 ligases such as NEDD4-1 [40] and CHIP [41] may also be influenced by AFP, which need to be further investigated. Meanwhile we found that miR-1236 inhibits the PI3K/Akt pathway by altering AFP expression. Our results show that miR-1236 inhibits the phosphorylation of AKT via decreasing AFP-mediated PTEN ubiquitination. Additionally, the over-expression of AFP can rescue the effect of miR-1236 on the phosphorylation of AKT. These results demonstrate that the PI3K/Akt pathway may be a mechanism through which miR-1236 regulates the development of HCC.

In conclusion, our results demonstrate that miR-1236 inhibits the proliferation, migration, invasion and VM through the down-regulation of AFP expression and inhibits the PI3K/Akt pathway via decreasing AFP-modulated PTEN ubiquitination (Figure 7). These findings may provide new insights into the mechanisms of HCC development and present potentially diagnostic or therapeutic strategies for HCC.

## MATERIALS AND METHODS

### Tissue Samples and Cell Lines

Twenty pairs of HCC tissues and adjacent non-tumor tissues confirmed by pathologists were collected from the Sun Yat-sen University Cancer Center. Informed consent was obtained from each patient, and all of the experiments were approved by the ethics committee of the institute.

The HCC cell lines QGY-7703 and SMMC-7721 were cultured in RPMI 1640 medium (GIBCO BRL, Grand Island, NY) supplemented with 10% FBS (fetal bovine serum) and 1% PS (100 units/ml penicillin, 100 μg/ml streptomycin). The Huh-7 and 293T cells were cultured in DMEM (GIBCO BRL, Grand Island, NY) with 10% FBS and 1% PS, and the HepG2 cells were cultured in MEM-a (GIBCO BRL, Grand Island, NY) with 10% FBS and 1% PS. All of the cells were maintained in a humidified incubator with 5% CO2 at 37 °C and transfected using LipofectamineTM 2000 reagent (Invitrogen, Carlsbad, CA).

### Plasmid Construction

To over-express miR-1236, the primary miR-1236 was amplified from genomic DNA of HepG2 cells and cloned into the pcDNA3 vector between the BamHI and EcoRI sites. To block the function of miR-1236, we purchased the 2′-O-methyl-modified antisense oligonucleotide of miR-1236 (ASO-miR-1236) and the scramble control oligonucleotides (ASO-NC) from the GenePharma (Shanghai, China).

The gene expressing AFP was amplified from the cDNA of HepG2 cells and it was cloned into the pcDNA3/Myc tag (pA3M1) and pcDNA3/Flag tag (pcDNA3-3flag) vectors between the EcoRI and XhoI sites. The primers for knocking down AFP were synthesized from GenePharma (Shanghai, China) and were annealed and cloned into the pSilencer2.1-neo vector (Ambion) between BamHI and HindIII sites.

The 3′UTR of AFP (containing the predicted binding sites for miR-1236) was amplified from the cDNA of HepG2 cells and then were cloned into pcDNA3-EGFP vector between the BamHI and EcoRI sites (downstream of EGFP). The mutant 3′ UTR of AFP (5 nucleotides were mutated in the miR-1236 binding sites) was amplified from the construct (pcDNA3-EGFP/AFP 3′UTR). All of the constructors were sequenced and primers are listed in Table 1

**Table 1:** The primers used in vector consructions

Name	Primer Sequence
pri-miR-1236 sense	5′-CGCGGATCCCTGGCCCTCACTTACCTC-3′
pri-miR-1236 anti-sense	5′-CCGAATTCCCATCTACATTCCAACTTGGAG-3′
ASO-miR-1236	5′-CUGGAGAGACAAGGGGAAGAGG-3′
ASO-NC	5′-CAGUACUUUUGUGUAGUACAA-3′
AFP sense	5′-ACTAGTAGCGGCCGCCAGTGTGCTG-3′
AFP anti-sense	5′-CTCCGGATCCTAGGTGACACTATAGAATAGG-3′
AFP-shR-Top	5′-GTACAAGGAAGTAAGCAAAATGTTCAAGAGACATTTTGCTTACTTCCTTGTA-3′
AFP-shR-Bottom	5′-AAAATACAAGGAAGTAAGCAAAATGTCTCTTGAACATTTTGCTTACTTCCTTGTAC-3′
AFP-3′UTR sense	5′-CACGGATCCAACTTGAGGCTGTCATTGC-3′
AFP-3′UTR anti-sense	5′-CGGAATTCGATAAGGAAATCTCACATAAAAGTC-3′
AFP-3′UTRmut sense	5′-AAATTACTTCAGGCCATCACAAGACAAAACG-3′
AFP-3′UTRmut anti-sense	5′-CGTTTTGTCTTGTGATGGCCTGAAGTAATTT-3′

### RNA Isolation and qRT-PCR Assay

Detailed procedures of RNA extraction and qRT–PCR were described in the previous study [21]. The specific primers used in this study are shown in Table 2.

**Table 2:** The primers used in qRT-PCR

Name	Primer Sequence
miR-1236 RT primer	5′-GTCGTATCCAGTGCAGGGTCCGAGGTGCACTGGATACGACCTGGAG-3′
U6 RT primer	5′-GTCGTATCCAGTGCAGGGTCCGAGGTATTCGCACTGGATACGACAAAATATGGAAC-3′
miR-1236 forward	5′-TGCGACCTCTTCTCCCCTTGTC-3′
U6 forward	5′-TGCGGGTGCTCGCTTCGGCAGC-3′
Reverse	5′-CCAGTGCAGGGTCCGAGGT-3′
AFP forward	5′-CACGGATCCAACTTGAGGCTGTCATTGC-3′
AFP reverse	5′-CGGAATTCGATAAGGAAATCTCACATAAAAGTC-3′
β-actin forward	5′-CGTGACATTAAGGAGAAGCTG-3′
β-actin reverse	5′-CTAGAAGCATTTGCGGTGGAC-3′

### Fluorescent Reporter Assay

To identify the direct target relationship between miR-1236 and the 3′ UTR of AFP mRNA, The QGY-7703 cells were cotransfected with pcDNA3/pri-miR-1236 or ASO-miR-1236 and the 3′ UTR of AFP or the mutant 3′ UTR of AFP in 48-well plates. The vector pDsRed2-N1 (Clontech) expressing RFP (red fluorescent protein) was transfected together with the above plasmids and used as the internal control standard. 48 h after transfection, the cells were lysed by RIPA buffer (150 mM NaCl, 1% Nonidet P-40, 1% Triton X-100, 1 mM MgCl2, 0.1% SDS, 10 mM Tris-HCl, pH8.0) and the fluorescent intensity of EGFP and RFP was measured with F-4500 fluorescence spectrophotometer (Hitachi).

### Western Blot Assay

Detailed procedure for western blot was described elsewhere [21]. Primary antibodies used in this study included AFP, PTEN, E-cadherin, vimentin, VE-cadherin, AKT, Myc and GAPDH were obtained from Saier Co. (Tianjin China). p-AKT antibody was obtained from Santa Cruze and the ubiquitin antibody was obtained from Sigma. The secondary antibody goat anti-rabbit and goat anti-mouse were obtained from Sigma. GAPDH was used as the endogenous control to normalize the expression level of interest proteins.

### *In vivo* Ubiquitination Assay

To detect the ubiquitination of PTEN *in vivo*, immunoprecipitation was used to isolate PTEN protein, and the ubiquitination level was detected by western blotting. The transfected 293T cells were treated by 10 μM proteasome inhibitor MG132 (Merck, Darmstadt, Germany) for 8 h before lysed by the ubiquitination lysis buffer (25 mM Tris-Cl PH 7.4, 150 mM Nacl, 1% NP-40, 10% glycerol, 1 mM DTT, 10 mM NaF, 20 mM NaVO3, 8 mM β-glycerophosphoric acid, 50 mM chloroacetamide) supplement with protease inhibitor cocktail (Roche, Indianapolis, IN, USA). The lysate was centrifuged for 10 min at 4 °C and next collect 100 μl supernatant acted as input. The left supernatant was incubated with myc or flag antibodies overnight at 4°C. The protein G was washed by ubiquitination lysis buffer twice and added to the lysate and antibody mixture, the final mixture was incubated at 4 °C for 6 h on rotary instrument. After incubation, centrifuge the mixture, and remove out the supernatant. The protein G left was washed three times by ubiquitination lysis buffer and added equivalent 2×loading buffer to the protein G, which was boiled for 5min. The interest protein and ubiquitination levels were detected by western blot.

### Colony Formation Assay

Detailed procedure for colony formation assay was described elsewhere [21].

### Migration and Invasion Assays

The 24-well Boyden chamber with 8 μm pore size polycarbonate membrane (Corning, Cambridge, MA) was used to analyze the migration and invasion of tumor cells. Detailed procedure was described elsewhere [19].

### Three-dimensional Matrigel culture (vasculogenic mimicry assay)

A 24-well cell culture plate was coated with 50 μl/well Matrigel (DB Biosciences), which was allowed to solidify at 37°C for 60 min. After the Matrigel had been solidified, the cell suspension (5×10^5^/well for HepG2 and 15×10^4^/well for QGY-7703) was plated into the well coated with Matrigel and incubated at 37°C for 24 h and then was observed and captured using microscope. We define the closed channel as one vasculogenic mimicry channel.

### Cell cycle analysis via flow cytometry

The transfected cells were plated to the 6-well plate in dulplicate and about 24h later when the cells became 60% confluent, replacing the culture medium by the serum free cluture medium for starvation. After 24 h starvation, one group of the cells were harvested and the other group was returned to complete culture medium for 24 h before harvest. The harvested cells were fixed in 95% (v/v) ethonal and stored at −60°C. Before tested, the stored cells were washed with PBS and then suspended in the propidium iodide (PI) staining buffer (PBS, 50μg/ml PI, 0.1mg/ml DNase-free RNase) for 30 min at 4°C. The samples were analyzed with FACS Calibur flow cytometer (DB Biosciences) and the FlowJo software (DB Biosciences).

### Tumor xenograft model in nude mice

The QGY-7703 cells were transfected with pri-miR-1236 or control vector. 3×10^6^ transfected cells were suspended by 100μl serum-free RPMI 1640 culture medium and were subcutaneously injected into the 6 week-old female nude mice in the flank. Mouse weight and tumor size were measured every other day after 8 days of injection. The tumor volume was calculated as follows: length×width^2^×1/2. All mice were killed 16 days after implantation. The tumors were isolated from the mice and stored at −80 °C. All studies were performed under the American Association for the Accreditation of Laboratory Animal Care guidelines for human treatment of animals and adhered to national and international standards.

### Immunohistochemistry

The tumors isolated from the mice were fixed in 4% formaldehyde for 24 h and sent to Tianjin Saier Co. for immunohistochemistry.

### Statistical analysis

Data are presented as the means ± SDs. Two-tailed Student's t tests were used for comparisons, and each experiment was performed at least three times. A p value less than 0.05 was considered significant (* p<0.05, ** p<0.01, *** p<0.001).
